# Case Report: A case of spinal muscular atrophy with extensively drug-resistant *Acinetobacter baumannii* pneumonia treated with nebulization combined with intravenous polymyxin B: experience and a literature review

**DOI:** 10.3389/fcimb.2023.1163341

**Published:** 2023-06-21

**Authors:** Bingqing Cao, Ling Cao

**Affiliations:** Department of Pulmonology, Affiliated Children's Hospital, Capital Institute of Pediatrics, Beijing, China

**Keywords:** polymyxin B, *Acinetobacter baumannii*, extensively drug-resistant, pneumonia, spinal muscular atrophy

## Abstract

Spinal muscular atrophy (SMA) is a neurodegenerative disease that results in progressive and symmetric muscle weakness and atrophy of the proximal limbs and trunk due to degeneration of spinal alpha-motor neurons. Children are classified into types 1–3, from severe to mild, according to the time of onset and motor ability. Children with type 1 are the most severe, are unable to sit independently, and experience a series of respiratory problems, such as hypoventilation, reduced cough, and sputum congestion. Respiratory failure is easily complicated by respiratory infections and is a major cause of death in children with SMA. Most type 1 children die within 2 years of age. Type 1 children with SMA usually require hospitalization for lower respiratory tract infections and invasive ventilator-assisted ventilation in severe cases. These children are frequently infected with drug-resistant bacteria due to repeated hospitalizations and require long hospital stays requiring invasive ventilation. In this paper, we report a case of nebulization combined with intravenous polymyxin B in a child with spinal muscular atrophy with extensively drug-resistant *Acinetobacter baumannii* pneumonia, hoping to provide a reference for the treatment of children with extensively drug-resistant *Acinetobacter baumannii* pneumonia.

## 1 Clinical data

### 1.1 General data

The child was a 4-year-old boy who was admitted to the hospital chiefly due to “postnatal hypotonia and intermittent asthmatic cough with dyspnea for 1 month.” The child, G1P1, was born by cesarean section at full term with postnatal hypotonia and delayed gross motor development and was diagnosed with spinal muscular atrophy (type I) at the age of 10 months after completion of genetic testing. One year prior, the child underwent tracheal intubation and was connected to an invasive ventilator for treatment for 26 days. The tracheal cannula was removed, and the child was discharged without a ventilator and was treated for the original disease with risdiplam orally for 8 months. One month before admission, the child developed a paroxysmal cough without obvious inducement, with more white mucous sputum aspirated, with fever, wheezing, and labored breathing. The child was hospitalized in a local tertiary hospital, and sputum culture showed *Acinetobacter baumannii*. Piperacillin sodium and tazobactam sodium, cefoperazone sulbactam sodium, tigecycline for anti-infection, fluconazole, and micafungin for antifungal infection were given successively. On the second day of hospitalization, the child’s dyspnea worsened. The dyspnea was relieved after the tracheal cannula was connected to an invasive ventilator for assisted ventilation. The child’s dyspnea worsened after removal of the tracheal cannula and ventilator twice, and the child was reintubated and connected to ventilator-assisted ventilation. The parents refused tracheotomy and transferred the child to our hospital. The child was admitted to the hospital with “bronchopneumonia and spinal muscular atrophy.”

The findings of the physical examination on admission were as follows: height 103 cm (P10–P25), weight 10 kg (<p3), respiration 40 bpm, heart rate 170 bpm, clear consciousness, slightly irritable, shortness of breath, positive nasal fan, and three depression signs. The patient had a bell-shaped thorax, coarse breath sounds in both lungs, scattered medium-fine moist rales, and wheezing sounds that were heard. There was no significant abnormalities in the cardiac and abdominal examinations; there was decreased muscle tone of the extremities, grade II muscle strength of the distal upper extremities, and grade I muscle strength of the proximal upper extremities and lower extremities. Tendon reflexes were not elicited, and both fingers were claw-shaped with contracture.

The routine blood results were as follows: WBC 8.21 × 10^9^/l, N 36.1%, L 54%, HGB 110 g/l, PLT 557 × 10^9^/l, CRP 2 mg/l. The blood gas analysis showed the following: P02 57.8 mmHg, PC02 44.1 mmHg, PH 7.413, HC03 27.6 mmol/l, S02 90.4%, and BE 3.1 mmol/l. The chest CT showed the following: there was a right lung upper lobe shadow, the bilateral lung lower lobe volume was decreased, a dense shadow was seen, a bronchial inflation phase was seen inside, there were bronchial gathering signs, there was an impression of pneumonia, and there was atelectasis of the bilateral lower lobes.

**Figure 1:**
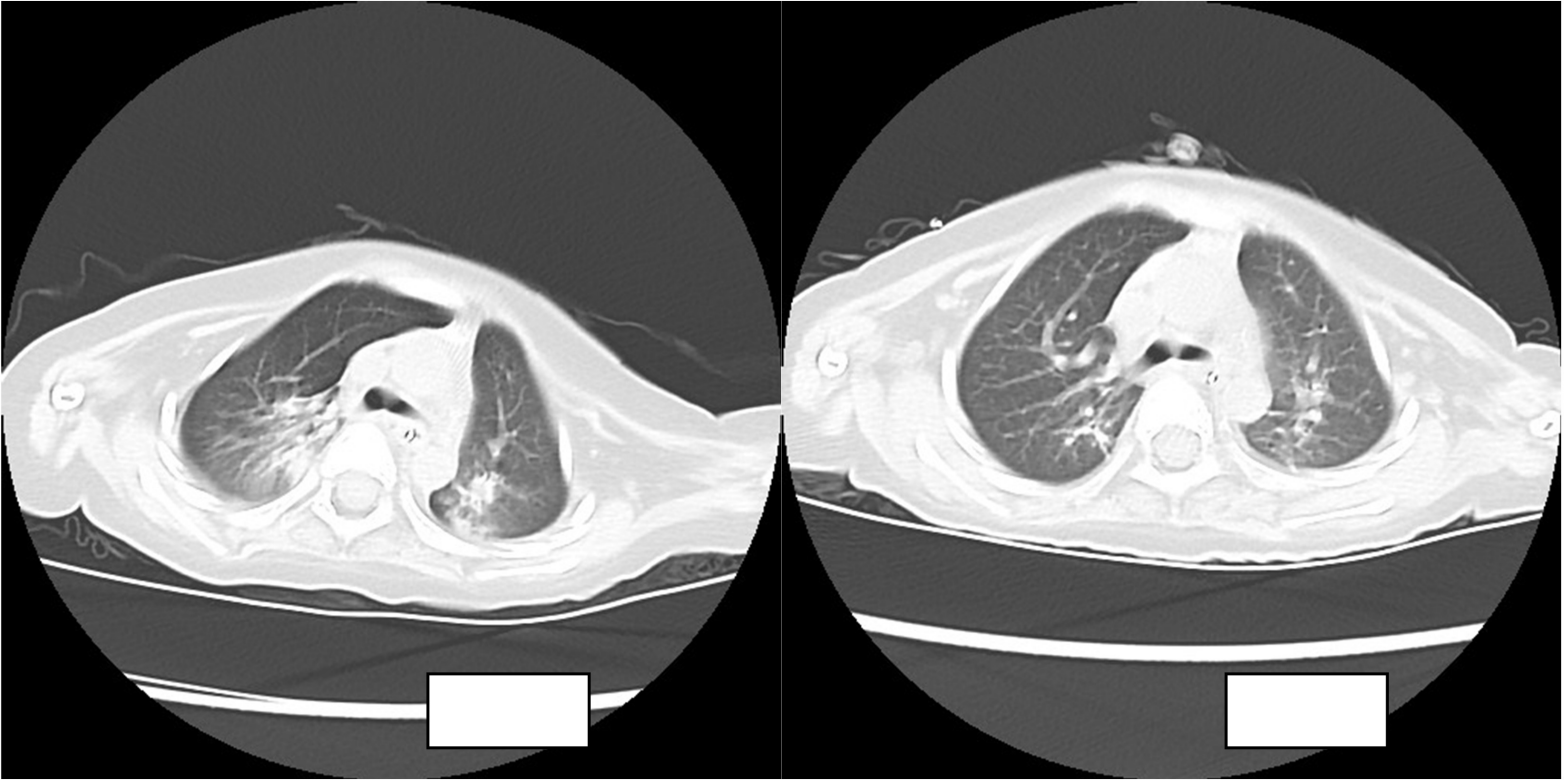
Comparison of the patient’s chest CT before and after treatment.

The child was admitted to the hospital with continuous invasive ventilator-assisted ventilation and was given cefoperazone sodium and sulbactam sodium ivgtt for anti-infection and intensive airway management. On the fourth day after admission, the bronchial lavage fluid was positive for *A. baumannii*, and sputum culture suggested a *S. aureus* and *A. baumannii* complex. Levofloxacin and tigecycline were added according to the drug susceptibility test. The child still had intermittent fever, and multiple bronchoalveolar lavage fluid and sputum bacterial cultures suggested the presence of the *Acinetobacter baumannii* complex, high- throughput gene detection tests on the bronchoalveolar lavage fluid suggested a high confidence level of the *Acinetobacter baumannii*. On the 44th day after admission, the child’s temperature was normal and his respiration was stable. Therefore, the tracheal cannula was removed, and the patient was changed to non-invasive ventilator-assisted ventilation. After maintenance for 4 days, the child was again fevered and intubated and connected to invasive ventilator-assisted ventilation due to dyspnea. The bacterial culture taken from the tip of the tracheal cannula on the 49th day of admission showed the following: *A. baumannii* complex, colistin-sensitive, cefoperazone, and sulbactam; the remaining isolates were resistant (including to tigecycline). **Table 1** shows our results. Tigecycline and levofloxacin were discontinued, intravenous polymyxin B combined with nebulization were added (intravenous administration: the first intravenous loading dose was 2.5 mg/kg (equivalent to 25,000 U/kg, 250,000 U/dose actually administered), and a maintenance dose at 1.25 mg/kg was given once after 12 h (equivalent to 12,500 U/kg/dose, 125,000 U/dose actually administered). For nebulization, 250,000 U/dose was dissolved in 5 ml saline; the solution was nebulized through a nebulizer device connected to a ventilator line, once/12 h) for anti-infectious treatment. The child’s guardian agreed to the treatment and signed an informed consent form. In the early stage of nebulization of polymyxin B, the respiratory rate increased and the blood oxygen concentration decreased. Therefore, a β2 agonist was inhaled 20 min before nebulization to reduce airway complications and oxygenated nebulization. The late stage of treatment went smoothly. After 5 days of polymyxin B treatment, the sputum culture was negative on two consecutive occasions and the reexamination of chest imaging showed improvement. The tracheal cannula was successfully removed on the 61st day of admission, and the patient was changed to non-invasive ventilator-assisted ventilation. Polymyxin B was discontinued after 14 days of use, and sputum culture showed no extensively drug-resistant *Acinetobacter baumannii*, with rechecked routine blood results as follows: WBC 4.89 × 10^9^/l, N 25.1%, L 62.4%, HGB 109 g/l, PLT 498 × 10^9^/l, CRP 0.46 mg/l; renal function results showed the following: BUN 5.83 mmol/l, Cr 12.2 µmol/l. The child was discharged uneventfully after a 75-day hospital stay. **Figure 1** illustrates the comparison of the patient's chest CT before and after treatment.

**Table 1:** Results of the bacterial culture taken from the tip of tracheal cannula on the 49th day of admission: *Acinetobacter baumannii* complex, solistin-sensitive, cefoperazone, and sulbactam; the remaining are resistant (including tigecycline).

Antibiotics	Results of the drug sensitivity tests	Minimal inhibitory concentration
Trimethoprim/sulfamethoxazole	Resistance	320
Piperacillin sodium/tazobactam sodium	Resistance	128
Tigecycline	Resistance	8
Cefoperazone/sulbactam	Intermediary	32 (≤16≥64)
Ticarcillin/clavulanic acid	Resistance	128
Colistin	Susceptible	0.5
Ceftazidime	Resistance	64
Ciprofloxacin	Resistance	4
Cefepime	Resistance	32
Doxycycline	Resistance	16 (≤4≥16)
Imipenem	Resistance	16
Levofloxacin	Resistance	8
Meropenem	Resistance	16
Minocycline	Resistance	16 (≤4≥16)
Tobramycin	Resistance	16

### 1.2 Literature review

The search terms “Polymyxin B,” “Acinetobacter baumannii,” “Extensively drug-resistant,” and “Pneumonia” were searched in PubMed and Web of Science, and the search terms “Polymyxin B,” “Acinetobacter baumannii,” “Extensively drug-resistant,” and “Pneumonia in children” were searched in the CNKI, SinoMed, and Wanfang databases, with the searching time being from database establishment until 01/09/2022.

The search did not include nebulization combined with intravenous polymyxin B for the treatment of childhood-associated pneumonia. Studies reporting nebulization of polymyxin B for the auxiliary treatment of pneumonia due to multidrug-resistant gram-negative infections have focused on adult patients. A recent prospective cohort study (Hasan et al., 2021) and a retrospective observational study in China (Zhou et al., 2021) showed that intravenous polymyxin B combined with nebulization therapy resulted in better clinical efficiency and microbial clearance, shortened the extubation time and ICU stay, and reduced the incidence of secondary infections without increasing the risk of renal damage. There are only a few clinical studies on the efficacy and safety of intravenous polymyxin B alone in the treatment of carbapenem-resistant gram-negative bacteria (CR-GNB) pneumonia in children, and these studies showed clinical efficacy rates of 47.8%–53.8%, microbial clearance rates of 30.8%–70.9%, in-hospital mortality rates of 7.3%–32.7%, and incidences of adverse events of 13.5%–27.3% (Liu et al., 2021; Jia et al., 2022; Wu et al., 2022).

## 2 Discussion

Spinal muscular atrophy (SMA) is a severe neuromuscular disorder due to a defect in the survival motor neuron 1 (SMN1) gene (Mercuri et al., 2018). Children with spinal muscular atrophy are easily complicated with upper and lower respiratory tract infections due to reduced cough and impaired secretion clearance, and according to statistics, the proportion of Chinese patients with type 1–3 SMA hospitalized with pulmonary infections ranges from 24.7% to 61.5% (Ge et al., 2012). When SMA patients present with pulmonary infections, in principle, pathogenetic examinations and infection assessments should be performed on a comprehensive case-by-case basis (Guo et al., 2016). Our department has analyzed the clinical characteristics of children admitted with spinal muscular atrophy and pneumonia and published related articles. All six children with long-term tracheal intubation and tracheotomy developed ventilator-associated pneumonia, and all of them had multidrug-resistant bacterial infections that required long-term use and replacement with multiple antibiotics based on the culture and drug susceptibility results, including four cases of *Acinetobacter baumannii* infection (Guo and Cao, 2020). Active treatment of pulmonary infections is important to achieve the early withdrawal criteria in children with tracheal intubation, with the aim of achieving a better long-term survival.

*Acinetobacter baumannii* has a strong ability to acquire drug resistance and clonal transmission, and multidrug resistant, extensively drug-resistant, and fully resistant *Acinetobacter baumannii* is endemic worldwide and has become one of the most important pathogens of nosocomial infections in China (Peleg et al., 2008). The most common site of nosocomial *A. baumannii* infection is the lung, and it is an important pathogenic bacterium of hospital-acquired pneumonia (HAP), especially ventilator-associated pneumonia (VAP) (Chen et al., 2012). The results of the 2021 CHINET China bacterial drug resistance surveillance (Hu et al., 2021) suggest that *A. baumannii* ranks fifth in clinical isolates in China, second only to *Klebsiella pneumoniae*. The resistance rate of *A. baumannii* was 71.5% to imipenem and 72.3% to meropenem. The resistance rate of carbapenem-resistant *Acinetobacter baumannii* to polymyxin B was only 0.5%. The results of the 2020 pediatric bacterial drug resistance surveillance by the ISPED (He et al., 2021) showed that the overall detection rate of carbapenem-resistant *Acinetobacter baumannii* (CRAB) was 33.5% (382/1140) and the overall resistance rate to multiple antibiotics was > 60%. In the face of such serious drug resistance, there are very limited therapeutic drugs available in the clinic, and polymyxins with specific antibacterial mechanisms have returned to the clinic with renewed attention (Infectious Diseases Committee of China Pharmaceutical Education Association et al., 2021). Polymyxin B (PMB) is often used as the last line of clinical defense in the treatment of extensively drug-resistant gram-negative bacillary infections (Lim et al., 2016).

The Management of Adults With Hospital-acquired and Ventilator-associated Pneumonia: 2016 IDSA/ATS Clinical Practice Guideline (Kalil et al., 2016) recommends that HAP/VAP caused by carbapenem-resistant bacteria be treated with intravenous infusion of polymyxins (colistin or polymyxin B) (strong recommended, moderate quality evidence) supplemented with inhaled colistin (weakly recommended, low quality evidence). In recent years, several additional national and international guidelines and expert consensus have recommended adjuvant polymyxin nebulization with intravenous polymyxin for patients with hospital-acquired pneumonia (HAP) or ventilator-associated pneumonia (VAP) caused by suspected or confirmed multidrug- resistant (MDR) or extensively drug-resistant (XDR) bacterial infections (Committee of Critical Care Medicine, China Society of Research Hospitals et al., 2019; Tsuji et al., 2019; Infectious Diseases Committee of China Pharmaceutical Education Association et al., 2021; Infectious Diseases Group of the Chinese Medical Association and Respiratory Diseases Branch, 2021). The recommended dosage and administration of polymyxin B sulfate nebulization is as follows: 250,000 to 500,000 U is dissolved in 5 ml of distilled water and nebulized with a conventional device, and a β2 agonist is inhaled 20 min before nebulization twice a day. The use of a vibrating mesh nebulizer is recommended.

Current studies on polymyxin B nebulization for the adjuvant treatment of pneumonia caused by multidrug-resistant gram-negative bacterial infections have mainly focused on adult patients. In the field of pediatrics, there are only a few clinical studies on the efficacy and safety of intravenous polymyxin B alone in the treatment of pneumonia in children with carbapenem-resistant gram-negative bacteria (CR-GNB), and there is a lack of data on the safety and efficacy of polymyxin B nebulization in pediatric patients. In addition to the guidelines and expert consensus, some studies have shown that nebulization of polymyxin B is effective and safe in pneumonia caused by infections due to resistant gram-negative bacilli for which intravenous administration is not effective (Pereira et al., 2007). Polymyxin B nebulization can reduce the course of respiratory-associated pneumonia caused by multidrug-resistant gram-negative bacillus infections and has low nephrotoxicity (Abdellatif et al., 2016). The efficacy and safety of polymyxin B nebulization have also been reported negatively, and Demirdal et al. (2016) showed that the efficacy of polymyxin B nebulization in combination with intravenous administration for the treatment of hospital-acquired pneumonia or ventilator-associated pneumonia caused by gram-negative bacilli infection did not differ statistically significantly from the efficacy of intravenous administration alone. A case of acute respiratory failure secondary to nebulization of polymyxin B in an asthmatic patient has been reported in the literature (Ma, 1982). In addition, the Expert Consensus on the Rational Use of Nebulization Therapy (2019 Edition) (Chinese Medical Association Clinical Pharmacy Branch, 2019) does not recommend intravenous preparations for nebulization because it may induce asthma or increase the risk of lung infection.

The child in this case had an underlying disease of spinal muscular atrophy, recurrent pulmonary infections requiring tracheal intubation, and invasive ventilator support, and the child also had long-term hospitalizations with a variety of advanced antibiotic therapies. The current pulmonary lesions were serious, and it was difficult to withdraw the ventilator. The child had an extensively drug-resistant *Acinetobacter baumannii* infection, the drug susceptibility results showed colistin sensitivity, and the remaining isolates were resistant (including tigecycline). After the clinicians and pharmacists codeveloped the treatment regimen, the dosage and administration that were recommended were according to the 2021 Multidisciplinary Expert Consensus on the Rational Clinical Use of Polymyxin Antibacterial Drugs in China. At present, there is no nebulization formulation of polymyxin B in China. In this case, the administration route of polymyxin B was nebulization in addition to an intravenous drip, and the treatment process went smoothly.

In this case, during there were no other complications, such as nephrotoxicity, hepatic damage, neurotoxicity, or skin damage, except for a mild decrease in transcutaneous oxygen saturation and increased heart rate at the initial stage of nebulization. The overall treatment was safe and effective, and the shortcoming of this case report is that the blood concentration of polymyxin B could not be monitored. The success of treatment in this case will provide a data reference for the optimal application of polymyxin B in pediatric patients with pulmonary infections. Please note that, since we only had one case, we were unable to confirm the effect of nebulization in the case, although the overall effect was good. Whether nebulization has an ideal effect remains to be discussed further.

## Data availability statement

The original contributions presented in the study are included in the article/supplementary material. Further inquiries can be directed to the corresponding author.

## Ethics statement

The studies involving human participants were reviewed and approved by Children’s Hospital, Capital Institute of Pediatrics, Beijing, China. Written informed consent to participate in this study was provided by the participants’ legal guardian/next of kin. Written informed consent was obtained from the participant/patient(s) for the publication of this case report.

## Author contributions

BC reviewed the literature and analyzed the case. LC supervised the findings of this work. All authors discussed the results and contributed to the final manuscript.
